# New Insights into the Effects of Several Environmental Parameters on the Relative Fitness of a Numerically Dominant Class of Evolved Niche Specialist

**DOI:** 10.1155/2016/4846565

**Published:** 2016-12-22

**Authors:** Anna Kuśmierska, Andrew J. Spiers

**Affiliations:** ^1^School of Science, Engineering and Technology, Abertay University, Bell Street, Dundee DD1 1HG, UK; ^2^Department of Industrial Microbiology and Biotechnology, Faculty of Biology and Environmental Protection, University of Łódź, Łódź, Poland

## Abstract

Adaptive radiation in bacteria has been investigated using Wrinkly Spreaders (WS), a morphotype which colonises the air-liquid (A-L) interface of static microcosms by biofilm formation with a significant fitness advantage over competitors growing lower down in the O_2_-limited liquid column. Here, we investigate several environmental parameters which impact the ecological opportunity that the Wrinkly Spreaders exploit in this model system. Manipulation of surface area/volume ratios suggests that the size of the WS niche was not as important as the ability to dominate the A-L interface and restrict competitor growth. The value of this niche to the Wrinkly Spreaders, as determined by competitive fitness assays, was found to increase as O_2_ flux to the A-L interface was reduced, confirming that competition for O_2_ was the main driver of WS fitness. The effect of O_2_ on fitness was also found to be dependent on the availability of nutrients, reflecting the need to take up both for optimal growth. Finally, the meniscus trap, a high-O_2_ region formed by the interaction of the A-L interface with the vial walls, was also important for fitness during the early stages of biofilm formation. These findings reveal the complexity of this seemingly simple model system and illustrate how changes in environmental physicality alter ecological opportunity and the fitness of the adaptive morphotype.

## 1. Introduction

Adaptive radiation requires organismal evolvability, ecological opportunities, and diversifying selection which create new niches in terms of physical space and new interactions between organisms and environment or allow empty ones to be colonised by adaptive lineages [1–4]. This process is directly relevant to the bacterial colonisation of natural and engineered environments, affecting final population sizes (productivity) and other ecosystem processes, host survival, efficiency, and output, and can be investigated in experimental evolution studies using simple microcosms in which biotic and abiotic factors and parameters can be manipulated [5–8].

**Figure 1:**
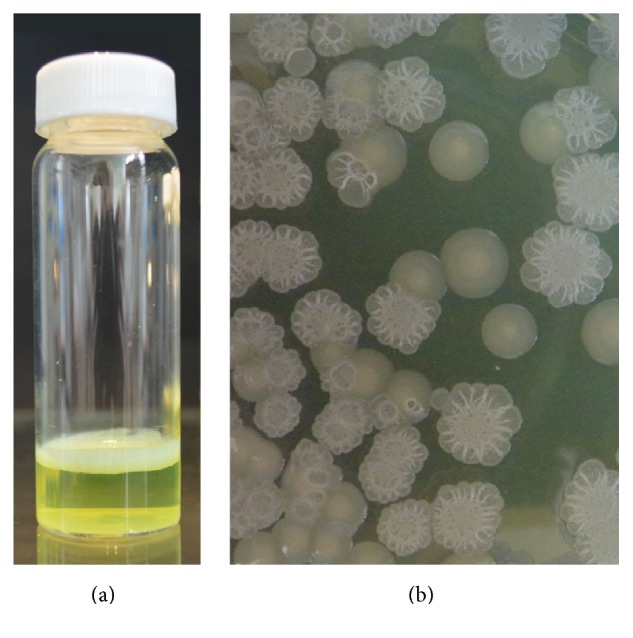
The Wrinkly Spreader is an adaptive morphotype which colonises the A-L interface in static microcosms. The WS phenotype is defined by (a) the formation of a robust, well-attached biofilm in static microcosms and (b) wrinkled colonies on agar plates which are readily distinguished from the smooth, rounded colonies produced by wild-type* P. fluorescens* SBW25.

One particularly successful system has used* Pseudomonas fluorescens* SBW25 populations grown in small glass vials containing nutritionally rich King's B medium [9]. These microcosms can be incubated statically to produce a heterogeneous environment with spatial structure and, in these,* P. fluorescens* SBW25 populations rapidly diversify over 3–10 days and accumulate mutants such as the Wrinkly Spreader. This class of adaptive mutant or morphotype [8] produces distinctive wrinkled colonies on agar plates and a robust, well-attached biofilm at the air-liquid (A-L) interface of static microcosms (sometimes referred to as pellicles, but see [10]) (Figure 1). It is the ecosystem engineering [3] by the early* P. fluorescens* SBW25 colonists that generates an O_2_ gradient in these microcosms and produces the ecological opportunity for Wrinkly Spreader colonisation of the A-L interface [11] (opportunity and niche are interlinked; see [8, 12, 13]). A range of mutations associated with diguanylate cyclases (DGCs) result in the Wrinkly Spreader (WS) phenotype through upregulation of c-*di*-GMP levels and the overproduction of partially acetylated cellulose (the primary extracellular polymeric substance (EPS) or matrix component) and attachment factor essential for colony morphology and biofilm structure [14–18]. This influential but simple experimental evolution model has been used to investigate aspects of adaptive radiation, including the importance of spatial structure (with high- and low-O_2_ zones) and resource competition (for O_2_ and nutrients), and of the emergence and maintenance of diversity [6, 8] which can be readily measured by determining the frequencies and final population sizes (productivity) of Wrinkly Spreaders and other morphotypes on agar plates (e.g., [9, 19, 20]).

Our interests are more focussed on developing a mechanistic explanation of how environmental parameters influence Wrinkly Spreader biofilm formation and fitness in this simple microcosm system. Colonisation of the A-L interface by the Wrinkly Spreader allows better access to O_2_ diffusing into the liquid column from the air above, providing a competitive fitness (*W*) advantage over the ancestral wild-type* P. fluorescens* SBW25 and other non-biofilm-forming mutants whose growth is O_2_-limited deeper into the microcosm [9, 11, 14, 21]. This advantage may reflect a significant change of physiology, as biofilm-isolated cells growing in the high-O_2_ zone can be differentiated from those recovered immediately below the biofilm by Raman spectral profiling [22] (*P. fluorescens* SBW25 can form a different type of biofilm when induced with Fe^3+^ which also provides a fitness advantage [23]). In the related pseudomonad,* P. aeruginosa* PA01, cells grow aerobically in A-L interface biofilms and, like the Wrinkly Spreader, these have a growth advantage over non-biofilm-forming competitors which are O_2_-stressed [24]. Furthermore, EPS production may be altruistic to cells as it pushes later generations into better O_2_ conditions above and helps suffocate non-EPS producers and cells lower down in the biofilm (ancestor's inhibition) [25]. O_2_ levels and the lack of physical disturbance (i.e., random knocks and vibrations) are probably the most important factors driving Wrinkly Spreader biofilm formation and longevity [11]. However, the advantage the Wrinkly Spreader has in static microcosms does not translate to other environments, as it is at a disadvantage in shaken microcosms and on agar plates where the WS phenotype is irrelevant and costly, and the ancestor effect may not be effective [9, 14, 21, 26].

As Wrinkly Spreaders are an adaptive morphotype in static microcosms (and because ecological opportunity and niche are interlinked), we expect that a “Goldilocks” effect should be apparent in these simple microcosms, and if environmental parameters differed, perhaps the Wrinkly Spreaders would not have the competitive fitness advantage they have over non-biofilm-forming competitors. In particular, there should be a tight link between the potential growth achievable at the A-L interface and WS fitness. However, the role of O_2_ is complex as the amount available, considered in terms of local concentration, during biofilm formation provides the ecological opportunity and reward for WS colonisation of the A-L interface, but O_2_ flux or supply (determined by the diffusion from the air above and uptake by the bacteria in the liquid column below) may have a more immediate impact on the developing WS biofilm and competitors. Furthermore, the importance of O_2_ also depends on nutrient levels and other factors, as if these became growth-limiting, O_2_ would no longer be a reward for colonisation of the A-L interface.

In this work, we manipulate several environmental parameters predicted to alter O_2_ levels in static microcosms in order to investigate how competition for this growth-limiting resource affects WS fitness in more detail and to further our understanding of the mechanisms underlying this commonly used model system.

## 2. Materials and Methods

### 2.1. Bacteria and Culture Conditions

The bacterial strains used in this work were wild-type* Pseudomonas fluorescens* SBW25 [27] and the archetypal Wrinkly Spreader (*P. fluorescens* SBW25* wspF* A901C) [14, 17]. Bacteria were cultured at 18–20°C in modified King's B (KB^*∗*^) medium (20 g proteose peptone (Oxoid, UK), 10 g glycerol, 1.5 g K_2_HPO_4_, and 1.5 g MgSO_4_ per litre with 1.5% (w/v) agar added for plates) and maintained at −80°C as 15% (w/v) glycerol stocks. The quantities of peptone and glycerol were reduced appropriately to produce media with 0.1x and 0.01x normal levels of nutrients. Standard microcosms were 30 ml Universal glass vials containing 10 ml KB^*∗*^ and were incubated statically or with shaking at 150 rpm using a Stuart S150 orbital incubator (Bibby Scientific Ltd., UK). Mineral oil (Fisher BioReagents, UK) overlays of 10–40 mm were added to microcosms to reduce O_2_ flux. Glass conical flasks and test tubes with 2.5–15 ml KB^*∗*^ were used to produce microcosms with different surface area/volume ratios. A 14 cm diameter Petri dish containing 100 ml KB^*∗*^ was used to assess biofilm formation over larger surfaces. Universal vials were filled to the brim with 37 ml and 39.5 ml KB^*∗*^ to produce microcosms with concave- (normal) and convex-shaped A-L interfaces, respectively. In order to minimise evaporation during the convex-concave experiments, microcosms were incubated in sealed containers with a dish of water to maintain humidity, and fresh medium was added every ~12 hr to maintain the convex shape (equal additions were also made to the concave microcosms).

### 2.2. Spectroscopy

A Spectronic Helios Epsilon spectrophotometer (Thermo Fisher Scientific, UK) was used for absorbance and optical density measurements using 1 cm optical-pathway cuvettes after zeroing using the appropriate sterile growth medium or solvent.

### 2.3. Preliminary Growth, Biofilm, and Oil Toxicity Tests

Preliminary tests were made to determine whether reduced-nutrient or oil-overlay microcosms impacted* P. fluorescens* SBW25 growth and WS biofilm formation. Microcosms were inoculated with 100 *µ*l aliquots of overnight static WS or shaken WT cultures as appropriate. Growth differences were assessed by optical density (OD_600_) measurements after 24 hr incubation. WS biofilm formation was assessed visually after 3 days. Oil toxicity was tested by comparing growth (OD_600_) between shaken KB^*∗*^ microcosms with and without 100 *µ*l oil after 24 hr incubation. For these, microcosms were left to stand for 30 min after vigorous mixing to avoid the transfer of emulsion or oil to the sample used for OD_600_ measurement.

### 2.4. Competitive Fitness Assay

The competitive fitness of the Wrinkly Spreader (WS) was determined relative to wild-type* P. fluorescens* SBW25 (WT) [21]. Replicate microcosms were inoculated with 100 *µ*l (per 10 ml) aliquots of a 1 : 1 mixture of overnight static WS and shaken WT cultures and incubated for 3 days before assay. The initial and final WS and WT viable cell numbers were determined by sampling the 1 : 1 mixture and 3-day microcosms after vigorous mixing, serial dilution, and enumeration on KB^*∗*^ plates. Competitive fitness (*W*) was calculated as the mean *W* = ln⁡[final WS/initial WS]/ln⁡[final WT/initial WT] [28]. Relative fitness (*W*/*W*_R_) is reported where R refers to the reference microcosms used for each assay.

### 2.5. Combined Biofilm Assay

Replicate microcosms (*n* = 8) were inoculated with 100 *µ*l (per 10 ml) aliquots of overnight static Wrinkly Spreader culture and incubated for 3 days before sequential assay to determine growth, biofilm strength, and attachment levels [29]. Briefly, biofilm strength (*S*) was first measured using the maximum deformation mass (grams) assay with small glass balls [30]. The microcosm contents were then transferred to another vial and, after vigorous mixing, used to determine growth (*G*) by optical density (OD_600_) measurements. The empty vial was then stained with Crystal Violet and the absorbance (A_570_) measurements of the eluted dye were used to determine the attachment levels (*A*) in the meniscus region [15]. It was necessary to wipe the outside rim of the vials to remove excess stain from the concave and convex microcosms before elution and measurement. Relative growth, biofilm strength, and attachment levels were calculated as for relative fitness (e.g.,* G*/*G*_R_, where R refers to the reference microcosms used in that assay).

### 2.6. Statistical Analyses

Data were analysed using JMP 12 statistical software (SAS Institute Inc., USA) and means with standard errors (SE) are reported. Normal quantile plots of the residuals were inspected with outliers removed if required and normality was assessed using the Shapiro-Wilk W goodness-of-fit test (*p* > 0.05). In large experiments, individual treatments were processed as single batches and, as a result, batch and treatment effects are combined. Comparison of means was by *t*-test and ANOVA, with post hoc comparisons made by Dunnett's test with control and Tukey-Kramer HSD tests.

## 3. Results and Discussion

### 3.1. The Experimental Microcosm System and the Determination of Fitness

As WS fitness is negatively frequency dependent [9], competition between Wrinkly Spreaders and the non-biofilm-forming* P. fluorescens* SBW25 competitor used in this work will vary depending on the relative starting ratio of strains. When the Wrinkly Spreader is rare, competition will primarily occur between the two strains and WS fitness will be high, but as the Wrinkly Spreaders become dominant, within-WS competition will increase and WS fitness will fall. In this work, we have balanced experimental workloads with the range of environmental parameters that could be investigated. We have chosen test WS fitness using a 1 : 1 starting ratio of strains at a standard cell density from which the Wrinkly Spreaders are expected to become rapidly dominant and produce a biofilm when conditions are favourable. Under similar starting conditions, changes in WS biofilm characteristics can be quantified [15, 29, 30], giving us confidence in linking WS fitness with biofilm formation. However, we note that alterations in these starting conditions will alter the dynamics of the system and possibly final outcomes. Finally, as in many of our experiments we observed relatively small fitness changes, we have chosen to report relative fitness (*W*/*W*_R_) using the appropriate reference microcosms, to reflect the magnitude of change rather than absolute competitive fitness (*W*) values.

### 3.2. WS Fitness Depends on the Ability to Dominate the A-L Interface and Reduce O_2_ Availability for Competitors

We predict that the competitive success of the Wrinkly Spreader in colonising the A-L interface of static microcosms is influenced by the physical size of the niche space available for WS colonisation compared to that available to the competitor. WS biofilms develop across the A-L interface before developing in depth and strength [15]. This suggests that the residents of the WS biofilm benefit by the biofilm spreading out across the A-L interface first to intercept O_2_ diffusion into the liquid column, and their growth subsequently becomes limited by reduced O_2_ and nutrient diffusion into the biofilm as it matures and thickens. As this area expansion-first strategy also has the effect of limiting O_2_ diffusion lower down into the liquid column, we do not expect that non-biofilm-forming competitors would be advantaged by deeper microcosms with greater nutrient resources as growth will still be O_2_-limited. We therefore expect to find that WS fitness will fall in microcosms with smaller A-L interface surface areas (SA) and fixed volumes (V) but will remain unchanged in microcosms with a fixed SA and increasing V.

**Figure 2:**
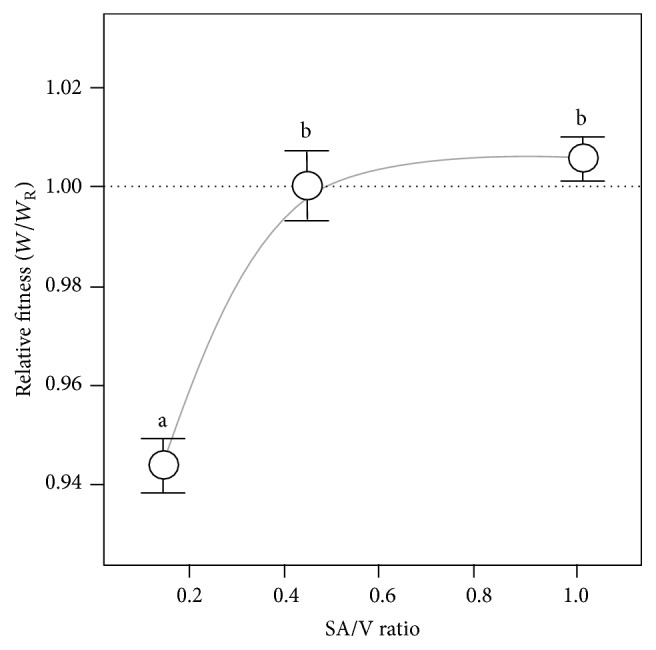
Expanding the physical niche available for colonisation increases Wrinkly Spreader fitness. Differently shaped containers were used to produce microcosms with a range of surface areas but with constant volumes (shown here as the SA/V ratio). Microcosms were incubated for 3 days before assay and the competitive fitness (*W*) of the Wrinkly Spreader was determined compared to the non-biofilm-forming* P. fluorescens* SBW25. Here, the relative fitness (*W*/*W*_R_) is provided where the reference microcosms with a SA/V ratio of 0.45 have a relative fitness of one (marked by the horizontal dashed line); the curve indicated here is illustrative only. Means ± SE are shown (*n* = 6), and means not linked by the same letter are significantly different (TK-HSD, *α* = 0.05).

We used a series of flasks, vials, and test tubes to produce microcosms with a range of SA/V ratios but with a fixed volume, from 0.15 to 1.02 cm^−1^. As the SA was reduced relative to V in these, the physical dimensions of the niche available for WS colonisation fell, and a small but significant decrease in relative fitness to 0.944 ± 0.006 (*W*/*W*_R_) was observed in the microcosms with a SA/V ratio of 0.15 cm^−1^ compared to the reference microcosms with a SA/V ratio of 0.45 cm^−1^, though not between the microcosms with SA/V ratios of 0.45 cm^−1^ and 1.02 cm^−1^ (TK-HSD, *α* = 0.05) (Figure 2). However, no significant differences in relative fitness were observed in fixed SA microcosms containing 2.5–15 ml KB^*∗*^ (TK-HSD, *α* = 0.05). These findings suggest that competitive interaction between the Wrinkly Spreaders and the non-biofilm-forming competitor is an inhibitory one that involves the Wrinkly Spreaders preventing competitors access to O_2_ which slows their growth and reduces maximal population size. We note that the Wrinkly Spreader can produce a biofilm in a 14 cm diameter container within three days, suggesting that biofilm formation is not limited by the need to be in close proximity to the vial walls. However, Wrinkly Spreaders may also have a maximal population size; as Wrinkly Spreader numbers grow in the maturing biofilm, only the top ~300 *µ*m layer of the biofilm remains O_2_-rich and lower regions of the biofilm become increasingly O_2_-limited [11]. In this situation, Wrinkly Spreaders start to compete with one another within the biofilm itself, with different WS mutants showing substantial morphological, metabolic, and fitness variation [17, 31–33].

We are, however, surprised that in deep microcosms the volume of media beneath the biofilm does not become increasingly attractive for colonisation by wild-type* P. fluorescens* SBW25 or a low-O_2_-adapted mutant, as we assume that this volume represents an ecological opportunity for an appropriately adaptive strain, as much as the A-L interface does for the Wrinkly Spreaders. Early work had suggested that the Fuzzy Spreader might be a bottom dweller [9], but this class of mutant has subsequently been shown to be a failed biofilm former which forms a sediment after physical disturbance [34]. In the case of* P. aeruginosa* PA01, supplementation with the alternative electron-acceptor nitrate facilitated the anaerobic growth of non-biofilm-forming competitors, suggesting that cells growing in the A-L interface biofilm face a trade-off between O_2_ and nutrient acquisition [24]. Similarly, in* Komagataeibacter xylinus* (formerly* Acetobacter xylinum*) A-L interface biofilms, growth is limited to a top layer of 50–100 *µ*m by O_2_ diffusion from above and nutrient diffusion from below [35]. Although* P. fluorescens* SBW25 is regarded by some as an obligate aerobe [34], this has yet to be established and we have not yet tried supplementing KB^*∗*^ with nitrate (or nitrite) to investigate the impact this would have on WS fitness or the colonisation of the low-O_2_ region of our microcosms.

### 3.3. Niche Quality Is Also Sensitive to O_2_ Flux

Earlier work had shown that O_2_ availability, considered in terms of local concentration, affected WS competitive fitness as WS competitive fitness (*W*) fell from 1.23 under normal O_2_ conditions to 0.12 when O_2_ levels were reduced to ~0.05% of normal levels [11]. However, O_2_ flux or supply (measured in terms of quantity/time/area) to the A-L interface is also likely to be a significant factor contributing to resource scarcity. Flux to the thin layer of liquid at the A-L interface where the WS biofilm is formed (the physical niche space) is dependent on the diffusion rate of O_2_ through air and water and on the uptake of O_2_ by bacteria at the A-L interface and lower down in the liquid column. Whilst flux differences affecting local O_2_ concentrations might alter growth rates, the total amount of O_2_ made available in these two regions will determine maximal population sizes over the 1–3 days in which WS biofilms form and our fitness assays are undertaken.

**Figure 3:**
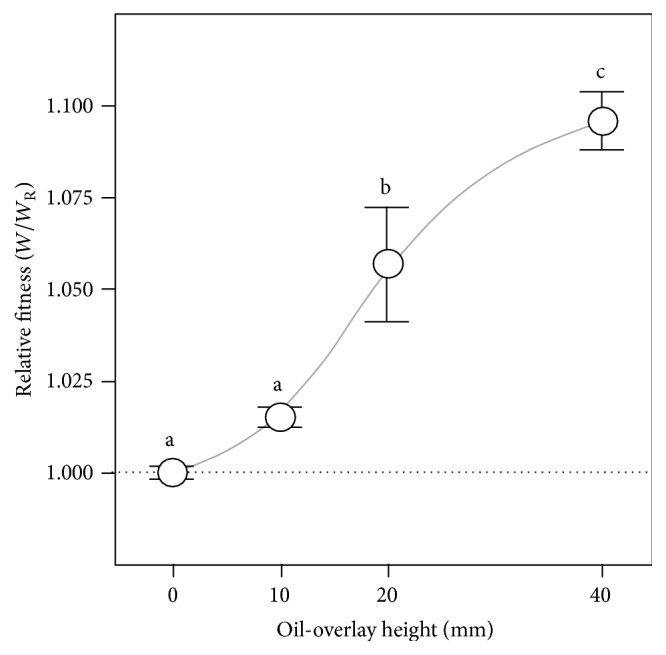
Reducing the quality of the niche available for colonisation by lowering O_2_ flux reduces Wrinkly Spreader fitness. Oil overlays of 10, 20, and 40 mm were used to reduce O_2_ flux or supply to the A-L or oil-KB^*∗*^ interface in static microcosms. Microcosms were incubated for 3 days before assay and the relative competitive fitness of the Wrinkly Spreader was determined compared to the non-biofilm-forming* P. fluorescens* SBW25. Here, the relative fitness (*W*/*W*_R_) is provided where the reference microcosms with no oil have relative fitness of one (marked by the horizontal dashed line); the curve indicated here is illustrative only. Means ± SE are shown (*n* = 6), and means not linked by the same letter are significantly different (TK-HSD, *α* = 0.05). The standard KB^*∗*^ microcosm (no oil) data are also shown in Figure 3.

We have used mineral oil overlays to reduce the diffusion of O_2_ to the oil-aqueous interface to investigate how changes in flux might affect WS fitness. An oil layer lying between the air and the KB^*∗*^ liquid column represents a diffusivity barrier to O_2_, as diffusion in light oil is lower than that in water and air (approx. 1 × 10^−9^, 2 × 10^−5^, and 2 × 10^−1^ cm^2^/s, resp., at 25–37°C) [36]. As a result, O_2_ flux or supply to the top of the KB^*∗*^ liquid column will be lowered by an intervening oil layer in an inverse proportional depth manner once the bacteria begin to take up O_2_. Preliminary experiments showed that the mineral oil used here had no toxic effect on* P. fluorescens* SBW25 growth in shaken microcosms (one-tailed *t*-test, *p* = 0.9993) and that WS biofilms formed immediately below the oil layer at the oil-aqueous interface of static microcosms containing 10–40 mm oil. However, although WS biofilms were produced in these oil-overlay microcosms, they were significantly reduced in terms of growth, strength, and attachment levels compared to the reference microcosms without oil (Table 1), suggesting that Wrinkly Spreader colonisation of the oil-aqueous interface was less successful. However, a small but significant increase in relative fitness up to 1.096 ± 0.008 (*W*/*W*_R_) was observed in microcosms with 20 and 40 mm oil overlays compared to the reference microcosms with no oil (TK-HSD, *α* = 0.05) (Figure 3), suggesting that the value of colonising the oil-aqueous interface increased with reduced O_2_ flux.

Although we initially struggled to resolve the apparent conflict between our oil-overlay fitness results with that of earlier work [11], we can propose a model which links both experimental results through an understanding of how local O_2_ concentrations and O_2_ flux to the A-L interface influence WS biofilm formation and fitness. At very low O_2_ levels and negligible flux, as provided by the sealed anaerobic bags used in the earlier experiments [11], the cost of biofilm formation by the Wrinkly Spreader was higher than the limited and short-term growth advantage achieved under these conditions, and, as a result, the Wrinkly Spreader had no fitness advantage over the non-biofilm-forming competitor (we calculate relative fitness of 0.12 (*W*/*W*_R_) in the low-O_2_ conditions compared to normal O_2_ levels from [11]). In contrast, although O_2_ flux was reduced in our oil-overlay experiments and impacted the growth and maximal population sizes of both the Wrinkly Spreader and the competitor, local O_2_ concentrations and the continued supply of O_2_ from the atmosphere allowed further growth and provided the ecological reward for WS biofilm formation. We might expect that if a higher-density starting inoculum was used, sufficient O_2_ might be removed from the KB^*∗*^ liquid column such that the reduced O_2_ flux could never supply sufficient additional O_2_ to allow the development of a biofilm. Similarly, a shorter incubation period should reduce WS performance as we expect that the maximum growth in Wrinkly Spreader populations occurs once the biofilm has been established in the high-O_2_ region of the microcosm, whilst longer incubation with a sufficiently high-O_2_ flux should improve matters whilst the biofilm develops in size and until within-WS competition begins to dominate.

### 3.4. The Importance of O_2_ to WS Fitness Is Linked to Other Growth-Limiting Factors Such as Nutrients

Bacterial behaviour is controlled by interacting regulatory systems to alter taxis towards energy sources or specific nutrients, O_2_, and so forth, the uptake of resources, and metabolism to maximise energy production and growth (e.g., [37–39]), and, in* P. fluorescens* SBW25 populations, diversification and final population sizes are dependent on nutrient levels [20, 40]. However, although O_2_ levels might be the dominating factor affecting growth, diversification, and fitness in normal static microcosms, we predict that the importance of O_2_ to WS fitness will decrease as other factors become progressively growth-limiting, and we have used microcosms with reduced-nutrient levels to test this prediction.

**Figure 4:**
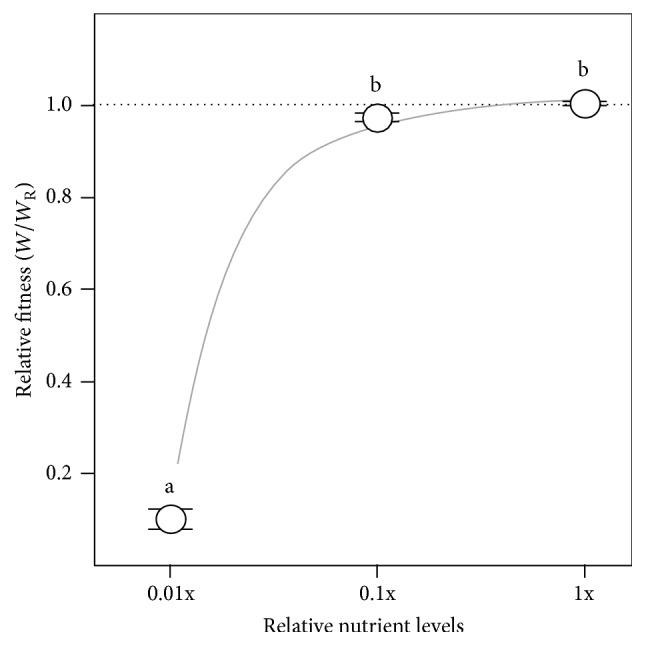
Reducing the importance of O_2_ reduces Wrinkly Spreader fitness. Nutrient levels were manipulated to reduce the importance of O_2_ in static microcosms. The nutrient component in KB^*∗*^ was diluted to produce microcosms with 0.01x and 0.1x normal nutrient levels. Microcosms were incubated for 3 days before assay and the relative competitive fitness of the Wrinkly Spreader was determined compared to the non-biofilm-forming* P. fluorescens* SBW25. Here, the relative fitness (*W*/*W*_R_) is provided where the reference microcosms with normal (1x) nutrient levels have relative fitness of one (marked by the horizontal dashed line); the curve indicated here is illustrative only. Means ± SE are shown (*n* = 6), and means not linked by the same letter are significantly different (TK-HSD, *α* = 0.05). The standard KB^*∗*^ microcosm data are also shown in Figure 2.

Preliminary tests were used to establish the notion that* P. fluorescens* SBW25 could grow in microcosms containing 0.1x and 0.01x normal nutrient levels, but not at 0.001x (Dunnett's test with control, *α* = 0.05). This is in agreement with other studies in which the impact of reduced-nutrient levels on the growth rate of* P. fluorescens* SBW25, carrying capacity, and time lag under similar conditions has been investigated [40]. Further tests showed that WS biofilms were produced under these conditions, but, like those in the oil-overlay experiments, they were significantly reduced in terms of growth, strength, and attachment levels compared to the reference microcosms containing normal nutrient levels (Table 1). Similarly, a large significant decrease in relative fitness to 0.096 ± 0.019 (*W*/*W*_R_) was observed in reduced-nutrient microcosms compared to the reference microcosms with normal nutrient levels (TK-HSD, *α* = 0.05) (Figure 4). This confirms that WS fitness is sensitive to nutrient levels but, more specifically, indicates that the effect of O_2_ on WS fitness depends on the availability of other resources which might become growth-limiting, revealing more complexity in this simple model system. From other work, both absolute nutrient levels and complexity are known to affect the adaptive radiation of* P. fluorescens* SBW25 populations, with reduced levels and single nutrient sources resulting in a lower frequency of Wrinkly Spreaders [19, 20, 40]. We can confirm this as, under the conditions used here, ~60% of the cells sampled after three days from microcosms with normal nutrient levels inoculated with wild-type* P. fluorescens* SBW25 were Wrinkly Spreaders; in contrast, none were observed in reduced-nutrient microcosms after the same time. However, we have not investigated how changing the inoculum size, growth rates, or final population sizes might also interact with reduced-nutrient levels to affect the appearance of Wrinkly Spreaders in radiating populations of* P. fluorescens* SBW25.

Collectively, the oil-overlay and reduced-nutrient experiments demonstrate the hidden complexity in this seemingly simple model system where WS fitness is affected predominantly by O_2_ levels, but also by O_2_ and nutrient interactions. We predict that, under sufficiently low O_2_ conditions, nutrient levels would begin to dominate, and, under optimal O_2_ and nutrient conditions, the physical dimensions of the microcosm would become important with large SA/V ratios favouring the Wrinkly Spreader and low ratios possibly selecting for low-O_2_-adapted mutants. Clearly, any response to altered resource levels or physical dimensions will be sensitive to the cell densities and relative numbers of Wrinkly Spreaders and non-biofilm-forming competitors used for inoculation as well as the length of incubation.

### 3.5. Concave A-L Interfaces and the Importance of the Meniscus Trap

**Figure 5:**
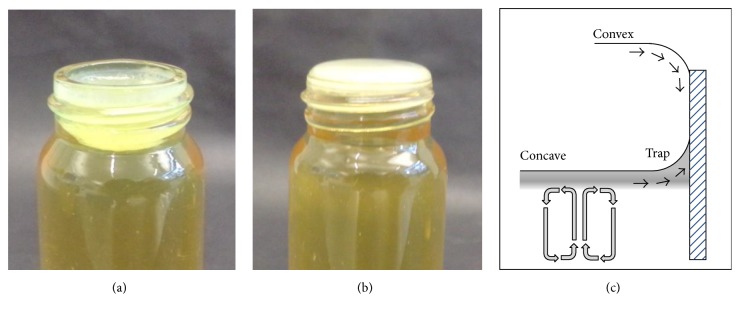
Wrinkly Spreaders colonise concave- and convex-shaped A-L interfaces. Microcosm vials were filled to the brim with KB^*∗*^ to produce (a) concave- and (b) convex-shaped A-L interfaces. A schematic of the upper regions of a microcosm (c) shows how the concave-shaped A-L interface interacts with the vial walls to produce the meniscus trap (which does not form with convex-shaped A-L interfaces). Only the top layer of liquid will have high-O_2_ levels (grey), and bacteria near the A-L interface are likely to be displaced radially towards the meniscus trap (black arrows) driven in part by bioconvection cells produced by bacterial motility.

The meniscus trap is a feature of the physicality of microcosms which we speculate aids WS biofilm formation and fitness. Although the progression of WS biofilm formation has yet to be recorded, it is likely that individual cells or rafts of cells first attach to the vial walls at the meniscus before growing outwards to cover the A-L interface. We expect that the acute angle formed at the meniscus by the air-liquid-solid surface (A-L-S) interface will form a high-O_2_ trap for cells (Figure 5) which might be recruited to the A-L interface by swimming motility, bioconvection cells, and penetration of the interface by a combination of cellulose, attachment factor, and surfactant expression [16, 23, 41–43]. This model is based on sessile drops where a hydrodynamic vortex is created by bacterial O_2_ taxis and the downward gravitational effect due to cell density which enhances O_2_ diffusion into the liquid and traps cells near the A-L-S interface [43].

**Figure 6:**
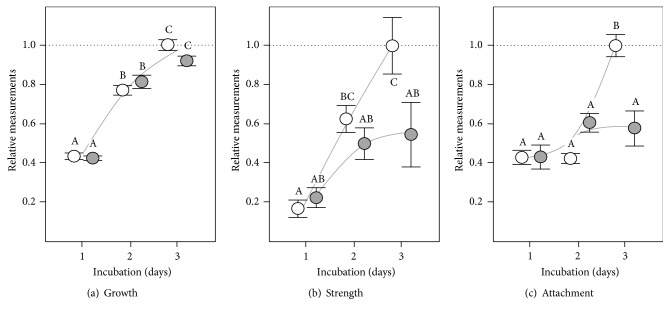
WS biofilm characteristics differed slightly between concave- and convex-shaped A-L interfaces. Microcosms with concave- (normal) and convex-shaped A-L interfaces were used to assess WS biofilm characteristics. KB^*∗*^ microcosms were incubated for 1, 2, or 3 days before assay. Relative measurements are shown for microcosm growth ((a), *G*/*G*_R_), WS biofilm strength ((b), *S*/*S*_R_), and attachment levels ((c), *A*/*A*_R_) where the 3-day-old concave reference microcosms have a value of one (marked by the horizontal dashed line); the curves indicated are illustrative only. Means ± SE are shown (*n* = 6) and means not linked by the same letter are significantly different (TK-HSD, *α* = 0.05).

**Figure 7:**
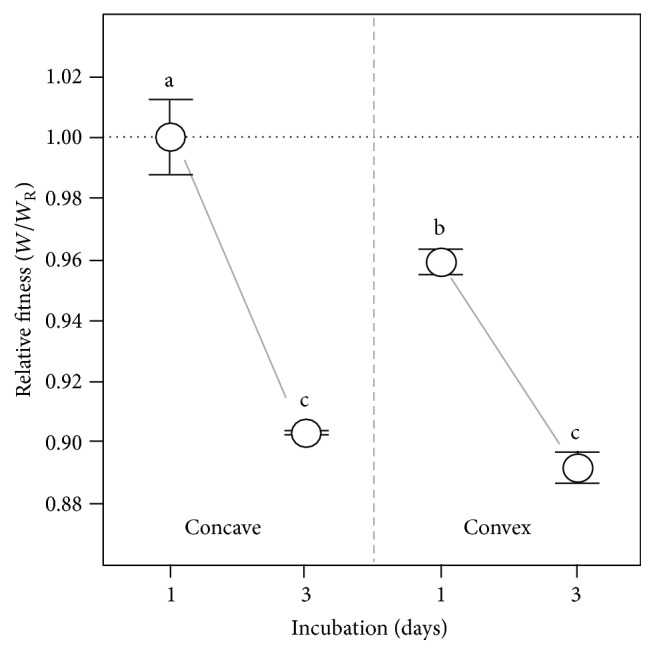
Concave A-L interface favours Wrinkly Spreader fitness. Microcosms with concave- (normal) and convex-shaped A-L interfaces were used to assess the importance of the meniscus trap in WS fitness. Microcosms were incubated for 1 or 3 days before assay and the relative competitive fitness of the Wrinkly Spreader was determined compared to the non-biofilm-forming* P. fluorescens* SBW25. Here, the relative fitness (*W*/*W*_R_) is provided where the reference microcosms with a concave-shaped A-L interface have relative fitness of one (marked by the horizontal dashed line); the curve indicated here is illustrative only. Means ± SE are shown (*n* = 6) and means not linked by the same letter are significantly different (TK-HSD, *α* = 0.05).

We overfilled microcosms to produce convex-shaped A-L interfaces lacking meniscus traps to investigate the impact this would have on WS biofilm formation and fitness (Figure 5). Preliminary experiments showed that WS biofilms formed in convex microcosms, though they were reduced in terms of growth, strength, and attachment levels compared to the reference microcosms with normal concave A-L interfaces (Table 1). Although relative microcosm growth and biofilm strengths increased similarly in concave and convex microcosms over three days of incubation, relative attachment levels in convex microcosms were found to be significantly reduced by ~0.6x compared to concave microcosms after three days (TK-HSD, *α* = 0.05) (Figure 6). This suggests that the meniscus trap supports better growth of attached cells and the early establishment of the WS biofilm in concave microcosms. A corresponding small but significant reduction in relative fitness to 0.959 ± 0.004 (*W*/*W*_R_) was found in convex microcosms compared to concave microcosms after one day (Figure 7), but not after the third day at which point relative fitness had dropped to ~0.9 (*W*/*W*_R_) in both types of microcosm (TK-HSD, *α* = 0.05), perhaps as the result of the unintentional damage of the biofilms caused by the daily additions of media required to maintain A-L interface shapes.

## 4. Conclusion

Experimental populations of* P. fluorescens* SBW25 in simple microcosms have proved to be a useful model system for investigating bacterial adaptive radiation and allowed mechanistic links to be made between mutation, the Wrinkly Spreader morphotype, and WS fitness advantage. Although this system is simple, our results provide new insight into the effects of several environmental parameters on the relative fitness of this numerically dominant class of evolved niche specialist. As such, the work represents an advance in our understanding of this influential model system, highlighting the way in which O_2_ and other factors interact, increasing complexity, and impacting WS fitness. Further manipulation of the system to enhance the development of low-O_2_ adaptive morphotypes colonising the liquid column below the WS biofilm would allow a more comprehensive understanding of the adaptive radiation of an ancestral genotype into interlinked niches with quite different selective pressures.

## Figures and Tables

**Table 1:** Wrinkly Spreader biofilm characteristics in modified microcosms.

Treatment		Relative microcosm growth (*G*/*G*_R_)	Relative biofilm strength (*S*/*S*_R_)	Relative attachment (*A*/*A*_R_)
Standard KB^*∗*^ microcosm	No oil, 1x nutrients	1.000 ± 0.051^a^	1.000 ± 0.167^a^	1.000 ± 0.081^a^

With oil overlays	10 mm	0.451 ± 0.026^b^	0.153 ± 0.024^b^	0.311 ± 0.026^b^
	20 mm	0.381 ± 0.017^b^	0.226 ± 0.035^b^	0.394 ± 0.030^b^
	40 mm	0.247 ± 0.011^c^	0.194 ± 0.046^b^	0.375 ± 0.044^b^

With reduced-nutrient levels	0.1x	0.408 ± 0.009^b^	0.194 ± 0.021^b^	0.535 ± 0.106^b^
	0.01x	0.117 ± 0.002^c^	0.113 ± 0.016^b^	0.343 ± 0.073^b^

Altered A-L interface shapes	Concave	1.000 ± 0.026^*∗*^	1.000 ± 0.144^*∗*^	1.000 ± 0.056^*∗*^
	Convex	0.918 ± 0.027	0.545 ± 0.166	0.577 ± 0.089

Microcosms were incubated for 3 days before assay. Relative means ± SE are shown (*n* = 6). The reference microcosms for the oil-overlay and reduced-nutrient assays were provided by the standard KB^*∗*^ microcosms. The reference microcosms for the altered A-L interface shape assays were provided by the concave microcosms. Comparisons of relative means were by assay and treatment. For the oil-overlay and reduced-nutrient experiments, means not linked by the same letter are significantly different (TK-HSD, *α* = 0.05). For the A-L interface shape experiment, means which are significantly different are indicated by *∗* (*t*-test, *p* ≤ 0.05).
